# Badnaviruses: The Current Global Scenario

**DOI:** 10.3390/v8060177

**Published:** 2016-06-22

**Authors:** Alangar Ishwara Bhat, Thomas Hohn, Ramasamy Selvarajan

**Affiliations:** 1 ICAR-Indian Institute of Spices Research, Kozhikode 673012, Kerala, India; aib65@yahoo.co.in; 2 UNIBAS, Botanical Institute, 4056 Basel, Switzerland; 3 ICAR-National Research Centre for Banana, Tiruchirapalli 620102, Tamil Nadu, India; selvarajanr@gmail.com

**Keywords:** badnavirus, integration, endogenous badnavirus, detection, distribution, host range, transmission

## Abstract

Badnaviruses (Family: Caulimoviridae; Genus: *Badnavirus*) are non-enveloped bacilliform DNA viruses with a monopartite genome containing about 7.2 to 9.2 kb of dsDNA with three to seven open reading frames. They are transmitted by mealybugs and a few species by aphids in a semi-persistent manner. They are one of the most important plant virus groups and have emerged as serious pathogens affecting the cultivation of several horticultural crops in the tropics, especially banana, black pepper, cocoa, citrus, sugarcane, taro, and yam. Some badnaviruses are also known as endogenous viruses integrated into their host genomes and a few such endogenous viruses can be awakened, e.g., through abiotic stress, giving rise to infective episomal forms. The presence of endogenous badnaviruses poses a new challenge for the fool-proof diagnosis, taxonomy, and management of the diseases. The present review aims to highlight emerging disease problems, virus characteristics, transmission, and diagnosis of badnaviruses.

## 1. Introduction

Plant pararetroviruses (Family: *Caulimoviridae*) contain eight genera with two distinct virion morphologies: *Caulimovirus* (10 species), *Soymovirus* (one species), *Solendovirus* (two species), *Cavemovirus* (two species), *Petuvirus* (one species), and *Rosadnavirus* (one species) have isometric particles, whereas members of *Badnavirus* (32 species) and *Tungrovirus* (one species) have bacilliform ones. All pararetroviruses contain a double-stranded DNA genome and replicate through an RNA intermediate, like retroviruses. However, in contrast to those, pararetrovirus genome integration is not part of their standard replication cycle. Instead, they accumulate as minichromosomes in the host nucleus. Illegitimate and usually fragmented pararetroviral integration occurs in different plants, as reported (Tabac TVCV-like) [1]; (banana BSV) [2]; (petunia PVCV) [3] (rice RTBV-like) [4], (potatoes*Sotu*EPRV) [5] and (tomato*Lyc*EPRV) [6]. Recently Geering *et al.* [7] described a new genus of Caulimoviridae called *Florendovirus*, members of which have colonized the genomes of large diversity of flowering plants including apple, citrus, cacao, grape, cassava, rice, potato, maize, papaya, soybean, tomato, *etc.* Integration occurs on average every million years [8].

**Figure 1:**
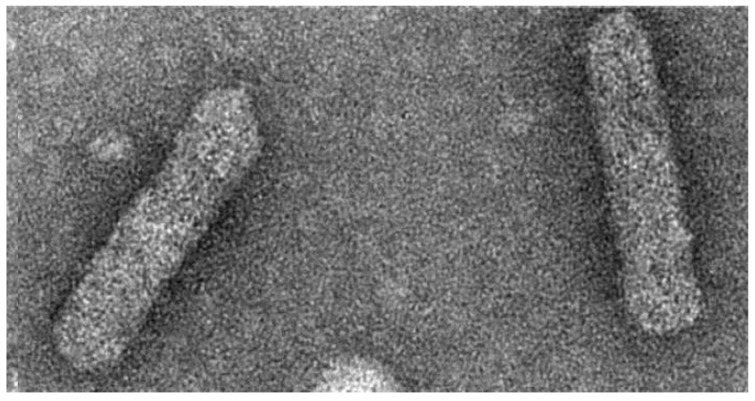
Electron micrograph of *Kalanchoe top spotting virus*, *Badnavirus*, Caulimoviridae. Crude sap preparation from infected Kalanchoe, negatively stained with 1% uranyl acetate. Photo: K. Richert-Pöggeler, copyright: Julius Kühn-Institut, Braunschweig.

Badnaviruses, the plant pararteroviruses of this review, infect a broad range of economically important crop plants all over the world [9,10,11]. The economic loss caused by the different species in various crops varies between 10% and 90%. The virions of badnaviruses are about 30 nm in diameter and vary in length between 120 and 150 nm, depending on the species (Figure 1) [12]. Virions are found in the cytoplasm and sometimes in vacuoles. The genome consists of a single circular molecule of double-stranded open circular DNA. It includes at least three open reading frames (ORFs) thought to be translated from the more-than-genome length RNA transcript. The complete genome is 7200–9200 bp long. Badnaviruses are also known to be present as integrated sequences in some host plant genomes and then referred to as endogenous badnaviruses [13,14]. The integration is assumed to have taken place by illegitimate recombination into host genomes, and their presence is not necessarily associated with infection. However, in some cases, these copies can give rise to systemic virus infection by recombination events, for instance induced by abiotic stress like *in vitro* tissue culture process [15,16] and interspecific crosses [17]. The presence of endogenous viruses poses a new challenge for fool-proof diagnosis, taxonomy, safe movement of germplasm, and management of diseases caused by badnaviruses.

## 2. Symptomatology, Host Range, and Transmission

**Figure 2:**
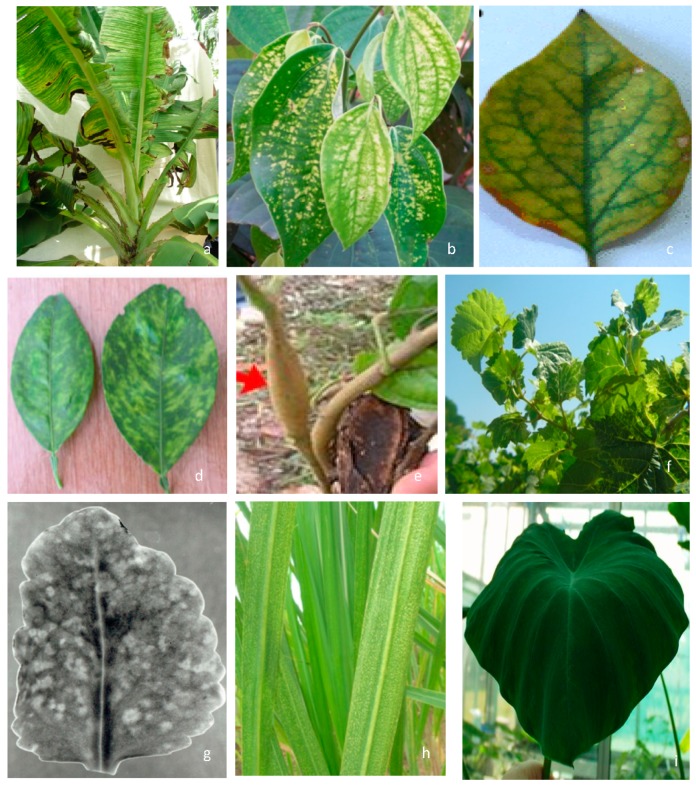
Symptoms of the diseases caused by badnaviruses. (**a**) chlorotic streaks in banana (Source: R. Selvarajan, ICAR-NRCB, Tiruchirapalli); (**b**)yellow mottle in black pepper (Source: A.I. Bhat, ICAR-IISR, Kozhikode); (**c**) chlorotic vein banding in bougainvillea (Source: V.K. Baranwal, ICAR-IARI, New Delhi); (**d**) yellow mosaic in citrus (Source: V.K. Baranwal, ICAR-IARI, New Delhi); (**e**) swollen shoot in cacao (Source: Wikipedia); (**f**) vein clearing in grapevine (Source: Wenping Qiu, Center for Grapevine Biotechnology, Missouri State University; (**g**) Kalanchoe top-spotting (Source: B.E.L. Lockhart, University of Minnesota); (**h**) streaks on sugarcane leaves (Source: R. Viswanathan, ICAR-SBI, Coimbatore); (**i**) umbrella-shaped leaf due to the infection of taro bacilliform virus (Source: Stephan Winter, DSMZ).

Badnaviruses are known to infect both monocots and dicots, though most of the species have a limited host range. In general, symptoms caused by badnaviruses are variable depending on the host, its cultivars, virus species, and environmental conditions. In most cases symptoms are mild to moderate. They include chlorotic mottle or necrotic streaks, deformation of leaves, and reduced internode length leading to stunting of plants (Figure 2). Asymptomatic nature of diseased plants and masking of symptoms during certain periods are common for most plants infected with badnaviruses. Symptoms’ re-emergence and severity increases when plants are subjected to abiotic stress, such as temperature shifts and depletion of nutrients. A majority of badnaviruses infect perennial hosts that are propagated vegetatively. Hence large-scale primary spread of badnaviruses occurs through vegetative propagation and a few, such as *Commelina yellow mottle virus* (ComYMV), *Kalanchoe top-spotting virus* (KTSV), *Piper yellow mottle virus* (PYMoV), *Cacao swollen shoot virus* (CSSV), and *Taro bacilliform virus* (TaBV), are also known to be seed-transmitted (Supplementary Materials Table S1). The secondary or horizontal spread of the majority of badnavirus species occurs through various mealybug species, while *Rubus yellow net virus* (RYNV), *Gooseberry vein banding associated virus* (GVBaV), and *Spiraea yellow leaf spot virus* (SYLSV) are transmitted semi-persistently by aphids. The first report of the occurrence of seed transmission (up to 40%) was in KTSV [18]. In CSSV-infected cocoa plants the virus was detected in every part of the pod and the virus was transmitted through seedlings [19]. Similarly, seed transmission of PYMoV was reported in black pepper [20,21] and of TaBV in taro [22].

## 3. Geographical Distribution

**Figure 3:**
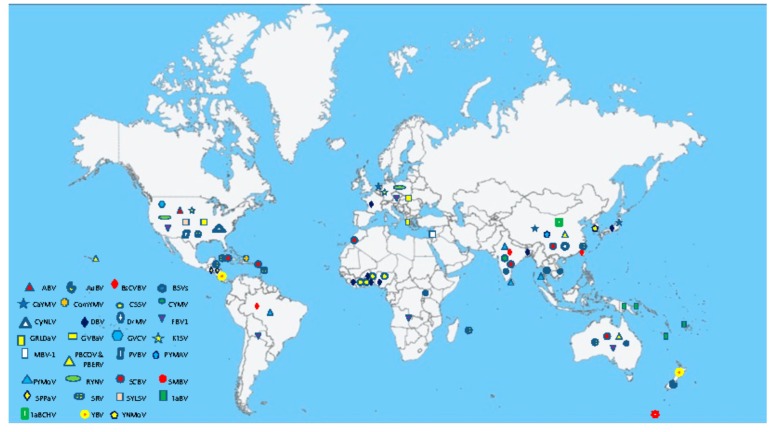
Worldwide distribution of badnaviruses. Details of acronyms used are provided in Supplementary Materials Table S1.

Badnaviruses are distributed in tropical and temperate regions of Africa, Asia, Australia, Europe, and South and North America (Supplementary Materials Table S1; Figure 3). The majority of species infect tropical and subtropical crops such as banana, black pepper, citrus, cocoa, sugarcane, taro, and yam. A few badnaviruses also infect plants in temperate climate zones, such as red raspberry, gooseberry, and ornamental spiraea.

## 4. Genome Organization and Replication Cycle

**Figure 4:**
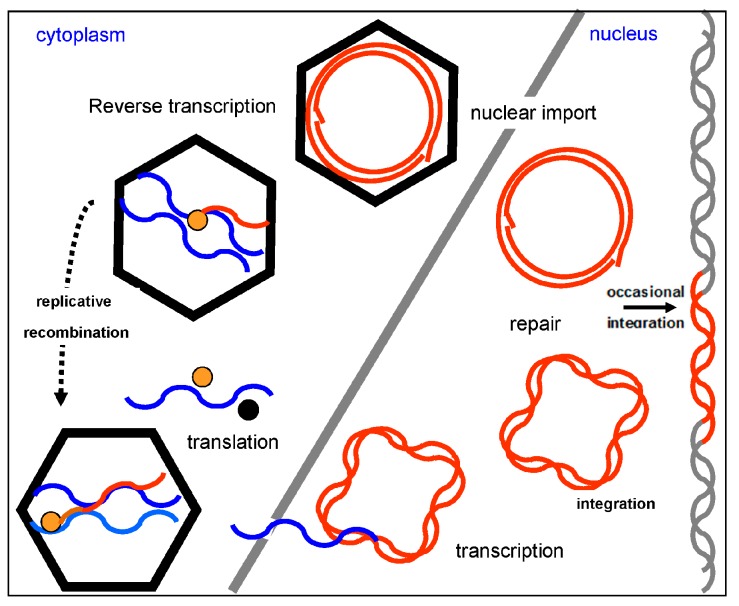
Replication cycle of plant pararetroviruses (Starting clockwise at 12 h): A virus particle containing open circular DNA has entered the cell. Its DNA is imported into the nucleus, where the gaps/overhangs are repaired to yield supercoiled dsDNA. Terminally redundant RNA is produced and transported into the cytoplasm. RNA copies are translated into virus proteins and/or packaged as two copies into virus particles. There reverse transcription occurs. The resulting open circular dsDNA reenters the replication cycle. In the lower left is shown how recombination can occur by template switching. On the right is shown how very rarely fragments of virus DNA incorporate into the plant genome, leading to endogenous viral sequences.

**Figure 5:**
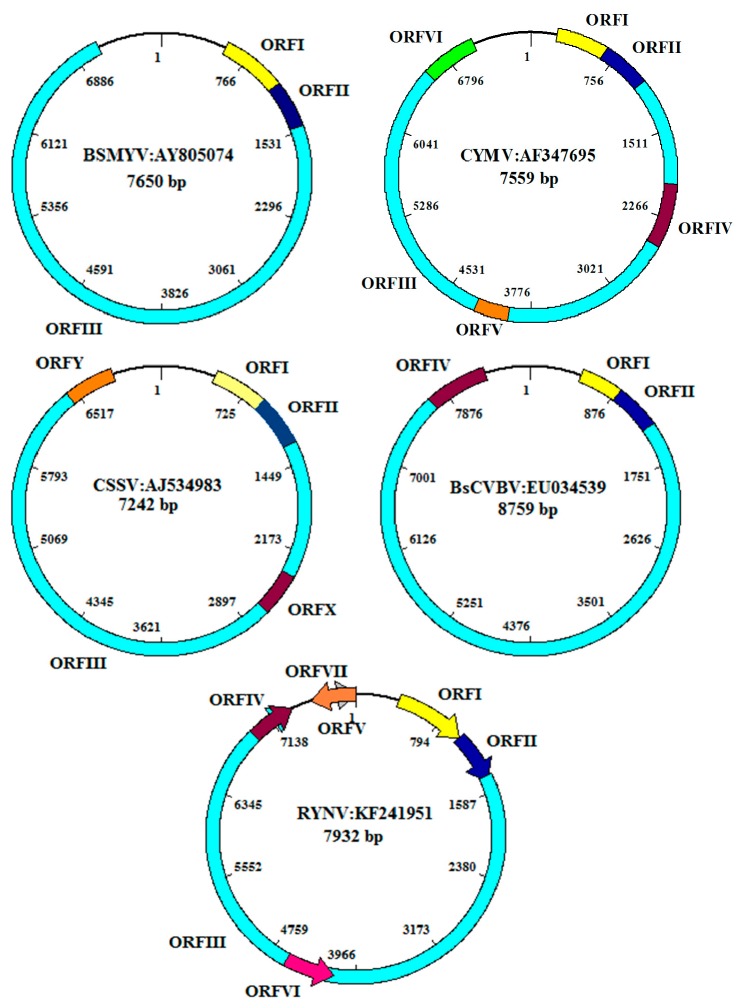
Genome organization of representative badnaviruses with varying number of open reading frames. BSMYV, *Banana streak MY virus*; BsCVBV, *Bougainvillea spectabilis chlorotic vein banding virus*; CSSV, *Cacao swollen shoot virus*; CYMV, *Citrus yellow mosaic virus*; RYNV, *Rubus yellow net virus*.

Replication of badnaviruses is thought to be similar to that of *Cauliflower mosaic virus* (CaMV) (Figure 4). The genomes of badnaviruses are 7.2 to 9.2 kb long relaxed circular DNA double strands with single-strand overlaps. Those are marks of the starts of plus- and minus-strand DNA synthesis by reverse transcription [23]. Reverse transcription occurs within the virus capsid and the resulting DNA is transferred to the nucleus, where the discontinuities are repaired and mini-chromosomes are formed by the association of the covalently closed, supercoiled DNA with histone proteins [24]. A 631 to 1177 bp long intergenomic region (Figure 5) includes a promoter and a polyadenylation signal. Mini-chromosomal DNA is then transcribed utilizing host-encoded DNA-dependent RNA polymerase II, creating a terminally redundant RNA that acts as pregenome and polycistronic messenger RNA. The redundancy is created by ignoring the polyadenylation signal on the circular DNA during the first encounter with the RNA polymerase and recognizing it at the second one [25]. In the final step of replication, the pregenomic RNA is converted back to dsDNA by the action of the reverse transcriptase. Synthesis of single-stranded, (−) sense DNA is primed by tRNA^met^ and of (+) sense DNA by purine-rich cleavage products left after RNase H digestion of the pregenomic RNA [11,23].

The ORF I of badnaviruses contain 399 to 927 bp depending on the species. In the type member ComYMV, ORF I is 602 bp long and translates into a 23 kD polypeptide, which was shown to be virion-associated [26]. The ORF II is the smallest, ranging from 312 to 561 bp. In CSSV, its product was identified as a nucleic acid-binding protein [27]; for ComYMV, it was shown also to bind to the virus capsid [11,26]. ORF III is the largest ORF, ranging in length from 5100 to 6000 bp. It encodes a polyprotein that is cleaved into four to five products (capsid protein, aspartate protease, reverse transcriptase, RNase H, and a putative cell-to-cell movement protein) [11,27,28,29]. The cleavage of this polyprotein into its functional subunits is achieved by aspartic protease [29]. The coat protein includes a “Cys” motif, as found in most reverse transcribing elements, and a second Cys motif (CX2CX11CX2CX4CX2C) found only in members of the genera.

Some badnaviruses have additional ORFs: PYMoV, Bougainvillea spectabilis chlorotic vein banding virus(BsCVBV), Dioscorea bacilliform SN virus (DBSNV), Grapevine Roditis leaf discoloration-associated virus (GRLDaV), TaBV, and Yacon necrotic mottle virus (YNMoV) have one [30,31,32,33,34,35]; CSSV and Pagoda yellow mosaic associated virus (PYMAV) have two [28,36]; Citrus yellow mosaic virus (CYMV) and Taro bacilliform CH virus (TaBCHV) have three [37,38,39]; and DrMV and RYNV four additional ORFs. Two of the latter are positioned on the antisense strand [40,41] (Supplementary Materials Table S1; Figure 5). It is not known whether these ORFs are expressed, nor what their function could be. The transcripts of plant pararetroviruses have a roughly 600 nt long highly structured leader, containing several short ORFs with the potential to form a large stem-loop structure, known to be inhibitory totranslation. Formation of this structure brings the first long ORF into the close spatial vicinity of a 5’-short proximal ORF that terminates 5–10 nt upstream of the stable structural element. This element is shunted by the scanning ribosome and leads to translation of the first long ORF [42,43]. The conservation of these elements suggests conservation of the ribosome shunt mechanism in badnaviruses as well [44].

Most badnavirus RNAs include three ORFs positioned solely on the sense strand, and encoding proteins of about 23, 15, and 216 kD (Figure 4). How are these ORFs translated from the polycistronic virus RNA? ORFs I and II have only weak or non-AUG start codons. For *Rice tungro bacilliform virus* (RTBV) it was shown that some scanning ribosomes initiate translation at those weak start codons, while others pass them by leaky scanning, finally initiating translation at ORFs II and III [45].

**Figure 6:**
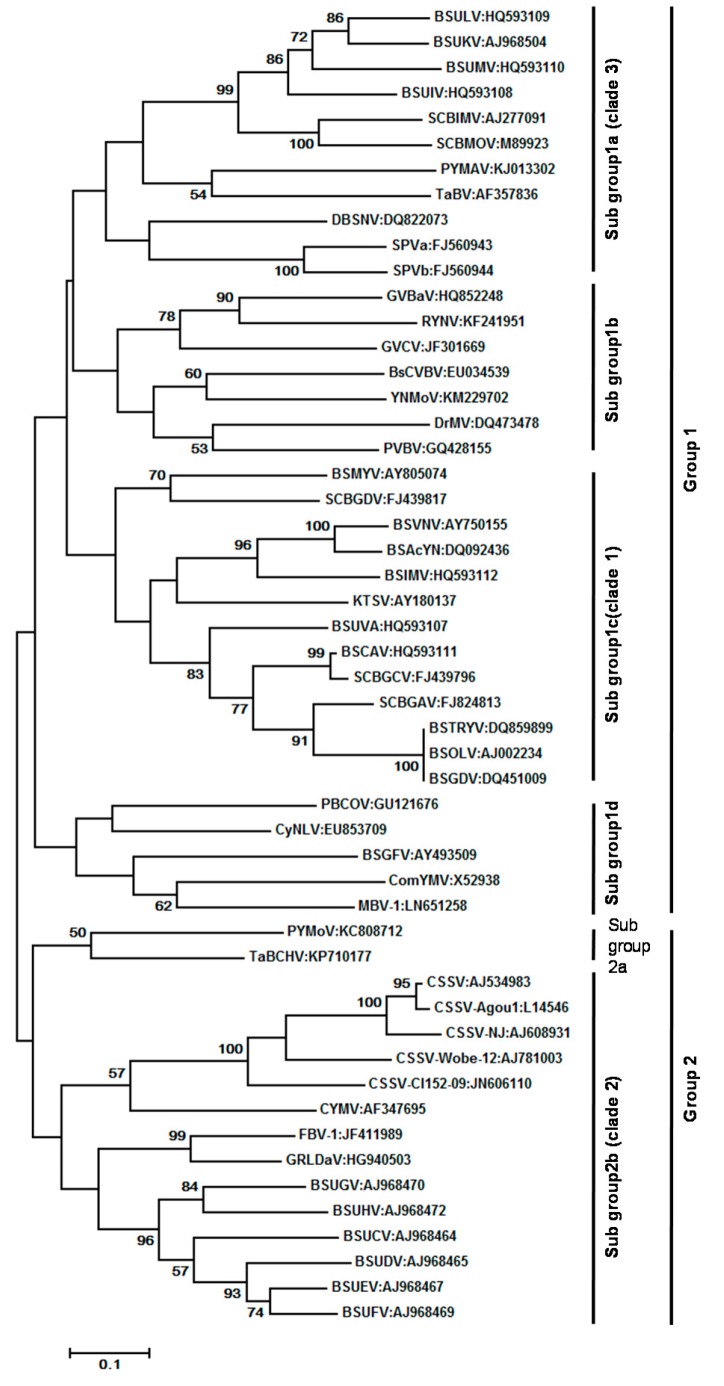
Maximum likelihood phylogeny of 52 badnavirus isolates representing different species based on alignment of a 540-bp fragment of the RT/RNase H viral region. Bootstrap values of 1000 replicates are given when >50%. The tree is drawn to scale, with branch lengths measured in the number of substitutions per site. Details of acronyms used are provided in Supplementary Materials Table S1. The GenBank accession numbers of sequences are given in parentheses. Clades 1, 2, and 3 shown in the figure areas per the phylogeny analysis [46].

At present, 70 complete genome sequences representing 28 recognized species and 20 complete genome sequences representing 16 putative species are available in the GenBank database (Supplementary Materials Table S1). CSSV has the smallest (7006 bp) and BsCVBV the largest (8759 bp) genome among confirmed species. Maximum likelihood phylogeny of 52 badnavirus isolates representing different species based on the alignment of a 540-bp fragment of the RT/RNase H viral region is furnished in Figure 6. The evolutionary history was inferred by using the Maximum Likelihood method based on the Tamura-Nei model. Evolutionary analyses were conducted in MEGA7. Initial tree(s) for the heuristic search were obtained automatically by applying Neighbor-Join and BioNJ algorithms to a matrix of pair wise distances estimated using the Maximum Composite Likelihood (MCL) approach, and then selecting the topology with superior log likelihood value. The 52 badnavirus sequences clustered into two major groups and group 1 could be divided into: subgroup 1a, which contained all *Banana streak virus* (BSVs) and *Sugarcane bacilliform virus* (SCBVs) of clade 3 published [46]; and subgroup 1b, which consisted of *Gooseberry vein banding associated virus* (GVBaV), RYNV, *Grapevine vein clearing virus* (GVCV), BsCVBV, YNMoV, DrMV, and *Pelargonium vein banding virus* (PVBV). The subgroup 1c comprised all the BSV species of clade 1 as per [46]. In contrast to earlier studies, the ComYMV and *Banana streak GF virus* (BSGFV) were grouped together in subgroup 1d. The other members of subgroup 1c are *Pineapple bacilliform CO virus* (PBCOV), *Mulberry badnavirus 1* (MBV-1), and *Cycad necrotic leafspot virus* (CyNLV). Group 2 was further divided into two subgroups. Sub-group 2a consisted of two species, *viz.* PYMoV and TaBCHV, and subgroup 2b consisted of members of BSVs, CSSV, and CYMV as reported in clade 2 of the previous study [46]. The other members of this subgroup were *Fig badnavirus 1* (FBV-1) and GRLDaV.

## 5. Nuclear Integration of Badnaviruses (Endogenous Badnaviruses)

Badnaviruses and other pararetroviruses, such as *Cavemovirus*, *Caulimovirus*, *Petuvirus*, *Solendovirus*, and *Tungrovirus*, are also present as integrated fragmented sequences in some host plant genomes. These are referred to as endogenous pararetroviruses (EPRVs) [6,13,47,48]. A separate genus, namely *Florendovirus* under the Caulimoviridae with 34 distinct species existing only as endogenous viral sequences, was reported [7]. The integration takes place by illegitimate recombination into host genomes, and their presence is not necessarily associated with infection. Endogenous sequences represent usually fragmented, linear forms of the original circular viral DNA. Methods such as *in situ* hybridization of labeled viral probes to the chromosome, Southern hybridization of the viral probe to the digested DNA of the host, immunocapture (IC)-PCR, rolling circle amplification (RCA), and finally plant genome sequencing have been employed by different workers to differentiate episomal and integrated forms of the virus. There are two forms of integrants: those that can form episomal viral infections and those that so far cannot. Integrants of three badnaviruses, *Banana streak OL virus* (BSOLV), BSGFV, and *Banana streak IM virus* (BSIMV) [8,49], as well as of several other plant pararetroviruses, such as *Tobacco vein clearing virus* (genus: *Cavemovirus*) [50] and *Petunia vein clearing virus* (genus: *Petuvirus*) [51], are known to generate episomal infections in certain hybrid plants in response to abiotic stresses such as temperature, water, nutrients, tissue culture, wounding, changes in day length, grafting, and breeding processes such as crossings. They reconstitute *de novo* infective particles possibly through a two-step intra-strand homologous recombination [52], thereby releasing a viral genome causing subsequent exogenous infections [46,53]. Integrants of other badnaviruses, such as certain BSVs (other than BSOLV, BSGFV, and BSIMV), DBV, FBV-1, KTSV, DrMV, and TaBV are not known to give rise to episomal infections, as only small portions of the viral genome, insufficient to give rise to complete virus sequences, are present [40,46,47,53,54,55,56,57,58].

The presence of endogenous viruses complicates approaches needed for diagnosis, taxonomy, safe movement of germplasm, and management of badnavirus diseases. Some endogenous viruses may just serve as components of plant genomes, while others could provide viral immunity [8,53,59].

## 6. Diagnosis and Cure of Badnaviruses

The symptoms caused by badnavirus infections are variable, and transmission to indicator plants is difficult due to the narrow host range of these viruses [60]. Visual inspection of symptoms is often unreliable as those are periodic and can be confused with the symptoms caused by other viruses. Lockhart [61] first reported the detection of BSV by serological methods. Given the heterogeneity of BSV isolates, the method was not generally successful. Hence, antiserum produced against a mixture of BSV isolates was used with some degree of success. Selvarajan *et al.* [62] reported ELISA and immunocapture PCR (IC-PCR)-based detection using polyclonal antiserum raised against virus-associated protein of BSMYV. Since the ORF II is distinct among the species of BSV, it was possible to develop species specific detection in ELISA. The serological and genomic heterogeneity of the badnaviruses is considered as the reason for low sensitivity of serological methods. Hence alternate methods have been devised by various workers. PCR has been used for the rapid, sensitive, and reliable detection of badnaviruses infecting different crops and vectors [58,63,64,65,66,67]. Real-time PCR and loop-mediated isothermal based assays have been reported for the detection of a few badnaviruses [68,69,70].

As mentioned above, endogenous badnaviruses pose a serious challenge to diagnostic testing. An assay that will detect episomal virus sequences may also detect integrated sequences due to the similarity in the sequences used to design primers. Thus to allow detection of episomal virus DNA alone, IC-PCR or multiplex immuno-capture polymerase chain reaction (IC-PCR) was used [64,71,72]. Rolling circle amplification (RCA) is a new promising method for the detection and amplification of the circular DNA genome of plant pararetroviruses [55,73]. RCA uses bacteriophage Φ29 DNA polymerase to amplify circular DNA molecules in a sequence-independent manner [74,75,76]. Since episomal badnavirus genomes are circular (as opposed to endogenous ones), RCA can discriminate between ds circular viral genome and endogenous viral sequences and thus overcome false positives, a common problem in standard PCR. RCA would also aid in the detection and identification of new badnaviruses. The RCA combined with RFLP was successfully employed in the detection of BSV species and FBV-1 [55,73,77]. A combination of immunosorbent electron microscopy (ISEM), IC-PCR, RCA, virus purification, Southern and *in situ* hybridizations, and complete sequencing of the virus genome may be required to elucidate the precise identification of the type of sequences present.

## 7. Virus Characterization

The genus contains several viruses of economic importance due to the severe diseases they cause: *Banana streak virus* (BSV), *Cocoa swollen shoot virus* (CSSV), *Citrus yellow mosaic virus* (CYMV), *Dioscorea bacilliform virus* (DBV), *Piper yellow mottle virus* (PYMoV), *Sugarcane bacilliform virus* (SCBV), and *Taro bacilliform virus* (TaBV). The International Committee on Taxonomy of Viruses (ICTV) [78] listed 32 species in the genus and another 20 species are awaiting recognition from the ICTV (Supplementary Materials Table S1). The ICTV has prescribed the guidelines for demarcation of species within the genus. The criteria for defining species are <80% identity nucleotide sequence) or <89% identity (amino acid sequence) in the conserved reverse transcriptase (RT)/ribonuclease H (RNase H) coding region [79].

### 7.1. Banana streak virus (BSV) (BSGFV, BSIMV BSMYV, BSOLV, BSUAV, BSUIV, BSULV, BSUMV, and BSVNV)

Banana streak disease of bananas and plantains worldwide is caused by a collection of *Banana streak virus* (BSV) species. Since its first report in Morocco in 1974, BSV has been reported from numerous countries in Africa, South America, and the Pacific and probably occurs worldwide wherever bananas are grown [60,80,81]. Symptoms of streak disease vary widely and are influenced by the cultivar, virus species, and environmental conditions. The typical symptoms include narrow, discontinuous chlorotic, and/or necrotic streaks that run parallel to the veins of the leaf lamina, and pseudostem splitting. In a few cases, internal necrosis, aberrant bunch emergence, fruit peel splitting, and necrotic fruit spots are also seen. Symptoms can reappear with periods of symptomless growth, temperature being considered as a factor in disease expression. Symptoms and virus load depend strongly on temperature. Enhanced symptom expression was seen when plants were continuously grown at 22 °C, but became indiscernible when plants were shifted to continuous growth at 28 °C to 35 °C. On the other hand, asymptomatic or mildly symptomatic plants grown at 28 °C to 35 °C significantly increased symptom severity and virus load nine months after transfer to 22 °C [82,83]. Potted hill banana (*cv.* Virupakshi, AAB) plants kept at 22 °C in an insect-proof glass house for six months to one year developed streak symptoms and BSGFV infection was confirmed by RCA and sequencing [84]. BSV infection of Cavendish banana (AAA genome) resulted in an 18-day delay in harvest, causing a 6% reduction in yield per annum in Australia [85]. The disease severity is greater among new banana and plantain breeding lines and micropropagated hybrids. Interspecific *M. acuminata* × *M. balbisiana* genotypes, including many newly created hybrids, showed BSV-infected propagates from originally virus-free source plants propagated by tissue culture, probably generated from endogenous sequences [16], and see below.

BSV is horizontally transmitted to bananas in a semi-persistent manner by mealybugs, of which *Planococcus citri* is the most prevalent, and vertically by mass-micropropagation—the main way to propagate banana plants or suckers.

Though initially all referred to as BSV [61], many distinct BSV species (BSVs) were distinguished based on sequence information and genetic and serological analysis [71,86,87,88,89,90], The first BSV isolate to be completely sequenced was from Nigeria; it contained 7389 bp, coding for three ORFs potentially encoding a protein of 20.8, 14.5, and 208 kD [91]. The isolate showed the highest sequence identity with SCBV. This isolate was later identified as a distinct species and came to be known as BSOLV (*Banana streak OL virus*). This was followed by complete genome sequencing of two Australian species, namely, BSMYV (*Banana streak MY virus*) and BSGFV (*Banana streak GF virus)* [71,87]. Harper *et al.* [88,89] reported that the variability of BSV in bananas from Uganda was extremely high and identified 13 new BSV species based on the DNA sequence of the conserved RT/RNase H region. Three other distinct full-length BSV sequences, namely *Banana streak VN virus* (BSVNV*) Banana streak IM virus* (BSIMV)*,* and *Banana streak Yunnan virus*, have been reported [86,90]. James *et al.* [92] proposed six additional new species from Uganda and Kenya, namely, BSUAV, BSUIV, BSULV, BSUMV, BSCAV, and BSIMV. So far, nine species have been recognized by the ICTV [78], namely, BSGFV, BSIMV, BSMYV, BSOLV, BSUAV, BSUIV, BSULV, BSUMV, and BSVNV (Supplementary Materials Table S1); additional ones are awaiting recognition [86,92]. The BSV species can be ordered into three clades: clade 1 includes several species causing streak disease worldwide; clade 2 encompasses *Musa* endogenous badnaviruses without episomal counterparts (see below); and clade 3 consists of all BSV species from Uganda except BSUAV [46,53].

**Figure 7:**
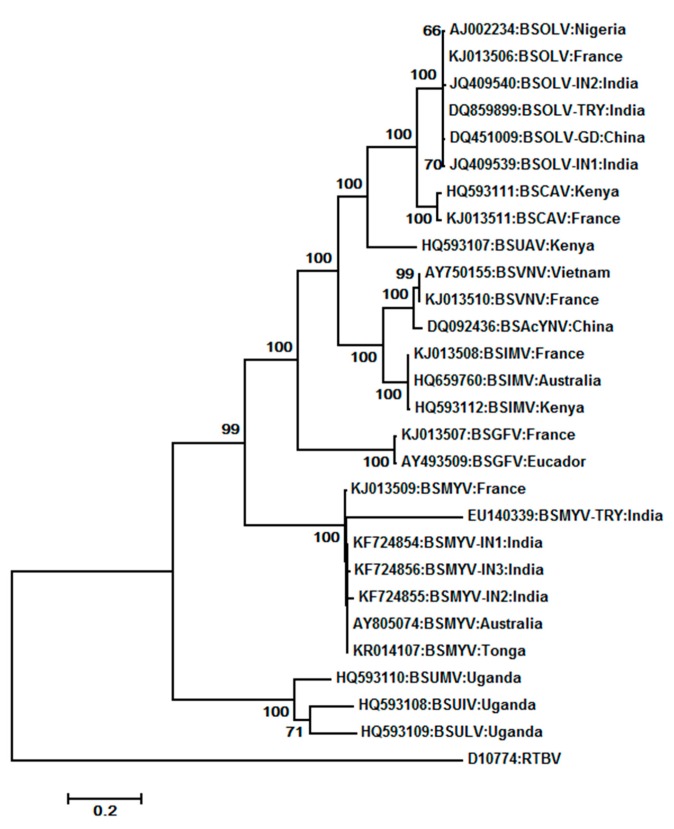
Phylogenetic clustering of 27 isolates belonging to different species of BSV based on amino acid sequence of open reading frame III. Acronyms used for viruses areprovided in Table S1. RTBV (*Rice tungro bacilliform virus*) was used as the outgroup.

We constructed a phylogenetic tree using the maximum-likelihood method using the MEGA 5.0 software by aligning of amino acid sequences of ORF 3 from the 27 complete genome sequences of BSVs available in NCBI GenBank with RTBV as the outgroup member (Figure 7). Different isolates of some BSV species clustered together, although their geographic origins were different. Three of four distinct species from Uganda (BSUMV, BSUIV and BSULV) were clustered together, while a Kenyan isolate, BSUAV, was clustered along with BSCAV isolates. BSOLV isolates from India, China, France, and Nigeria clustered together, as did BSMYV isolates from India, Australia, France, and Tonga.

Several BSVs, called endogenous BSV (eBSV), became part of the genetic material of their host species by endogenization of ancestor badnaviruses. The hosts are several *Musa* A (*M. acuminata*), B (*M. balbisiana*), and S (*M. schizocarpa*) species [93]. At this time, eBSV is the common name for endogenous viral sequences corresponding to a known episomal BSV. Only BSOLV, BSGFV, BSIMV, and BSMYV have an eBSV counterpart at this time [14]. These eBSVs are only present in the *M. balbisiana* genome (B genome). The other endogenous badnavirus sequences described [89,93], named by Geering *et al.* [93] as BEV (banana endogenous badnavirus), have not had an episomal counterpart identified so far. They likely correspond to old integrations events of ancestral viruses (the grand-grandfather of BSV) and are widely distributed among *Musa* genomes [46,53,94,95].

It was demonstrated by double target *in situ* hybridization integration of BSOLV sequences into metaphase and pro-metaphase chromosomes of the banana cultivar, Obino l’Ewai at two loci. *in situ* hybridization to stretched DNA fibers indicated that one of them is approximately 150 kb long, whereas the other is estimated to be 50 kb long [47,96]. At each locus, eBSV sequences are interrupted by unrelated *Musa* sequences. Sequence data revealed disrupted and truncated eBSVs with deletions, insertions, duplications, or inversions. Analysis of badnavirus integrants in *M. acuminata* showed their distribution among all 11 chromosomes [94]. All the badnavirus integrants appear fragmented and highly re-organized, with a size ranging from 100 bp to 18 kbp. Based on evolution rates and selective pressure on BSV and BEV sequences, Gayral and Iskra-Caruana [95] reported that there were at least 27 independent integration events that occurred after the divergence of three banana species, indicating that viral integration is a relatively frequent phenomenon. Illegitimate and homologous recombination is the primary mechanism for integration. They observed that the majority of the viral integrants in banana are defective as a result of pseudogenization driven by evolution of the host genome. However, three eBSVs existing in the genome of *M. balbisiana* are infective [8,49,97] and contain complete but mostly rearranged viral genomes. The rescue of infective viruses from eBSVs is reported only for BSOLV, BSIMV, and BSGFV. This occurred via activation by tissue culture, hybridization, or temperature differences in newly created banana interspecific hybrids [15,16,97]. A homologous recombination pathway for this event is proposed [52]. However, an alternative pathway of virus reconstitution is template switching during reverse transcription from fragmented RNA.

Infective eBSVs constitute an extreme case of parasitism, as well as a newly described strategy for virus vertical transmission [98]. The activated BSV particles can even be transmitted by mealybugs [99]. Non-activated integrated sequences of other eBSVs and BEVs are fragmented, rearranged, and have inactivating mutations. They are therefore replication defective and hence non-infective [93,95].

Banana is vegetatively propagated either by tissue culture or by production of suckers. Hence accurate diagnostic methods are necessary for identification of virus-free material. The presence of high levels of serological variability between BSV species and the presence of eBSV sequences make serological and PCR-based diagnosis difficult. For banana plants that contain ‘B’ genome and harbor both episomal and activatable endogenous BSVs (BSOLV, BSIMV, and BSGFV), use of RCA and PCR are needed, while for other banana plants, only RCA could be used to distinguish between endogenous and free viral genomes. Development of real-time PCR for the rapid detection of episomal BSOLVand BSMYV was reported [100,101,102,103]. Multiplex immunocapture PCR (M-IC-PCR) and rolling circle amplification (RCA) have been developed for the detection of BSV genomes [72,73,104]. RCA was more sensitive than PCR and serology-based methods in the detection of BSV [77]. Selvarajan *et al.* [62] developed serological-based detection of BSMYV using the recombinant virus-associated protein encoded by ORF II, which is very specific to each species of BSV. Helliot *et al.* [105] reported cryopreservation for the elimination of BSV from banana *cv.* Williams (AAA, Cavendish subgroup where no activatable BSV integrant is reported), where excised meristematic clumps were cryopreserved through vitrification using PVS-2 solution. The frequency of BSV elimination was 90% through cryopreservation, compared to 76% obtained through conventional meristem culture.

### 7.2. Bougainvillea spectabilis chlorotic vein-banding virus (BsCVBV)

Bougainvillea chlorotic vein-banding disease caused by *Bougainvillea spectabilis chlorotic vein-banding virus* (BsCVBV) was first reported in Brazil in 2001 and subsequently from Taiwan and India [106,107,108]. The diseased bougainvillea plants developed symptoms such as mottling, chlorosis, vein-banding, leaf distortion, and stunting. Symptom variability of different cultivars in Taiwan was reported [109]. The virus is transmitted through bud grafting. It was characterized by electron microscopy, which showed typical bacilliform particles, and by sequencing of a portion of ORF 3 revealing similarities with known badnaviruses. The complete genome contains 8759 bp with four ORFs [34] (Figure 5). Two distinct badnaviruses infecting *B. spectabilis* in two different locations of India and with distinct symptoms (severe yellow mosaic and chlorotic vein banding, rsp.) could be distinguished by sequencing of the RT/RNaseH region [108].

### 7.3. Cocoa swollen shoot virus (CSSV)

*Cocoa swollen shoot virus* (CSSV), which infects cocoa (*Theobroma cacao*), was first discovered in the eastern region of Ghana in 1936. It causes a devastating disease affecting cacao cultivation of most West African countries [110]. The disease is characterized by swelling of roots and the stem and leaf turning red [111]. Both virulent and mild strains of CSSV are known to occur, with severe strains killing cocoa plants within two years [112]. The recent and unique colonization of cacao in Africa likely due to a host-shift, and the high diversity of the virus in the main countries where the disease is reported (Côte d’Ivoire, Ghana, Togo) are the reasons for the severity of the disease. The virus has a restricted host range. It is not transmitted mechanically, but semi-persistently by *Planococcoides njalensis*, *Planococus citri*, and at least 12 other species of mealybugs [113]. The dissemination of the disease is mainly due to the use of infected plants (for long distance), mealybug-mediated transmission (for short distance), and newly created plantation in the primary forest areas. Recently, seed transmission was reported [19]. CSSV was found in all parts of cocoa seeds and seedlings obtained from CSSV-infected plants. Although CSSV was also detected in pollen, the virus could not be transmitted through cross-pollination [114].

The first complete genome sequencing of CSSV showed that it contains 7161 bp with five ORFs namely ORF I, II, III, X, and Y encoding proteins of 16.7, 14.4,211, 13, and 14 KDs, respectively, and an intergenic region [28] (Figure 5). ORF II of CSSV was shown to be a nucleic acid binding protein, while the functions of ORF I, X, and Y are unknown. ORF III encodes the standard badnavirus polyprotein [27,28]. Oro *et al.* [115] reported the molecular diversity of CSSV based on 120 sequence comparisons of the first part of ORF III and distinguished three groups. Complete genome sequence of five strains of CSSV originating from Togo and Ghana revealed the existence of up to 29% variability among different geographical isolates [116]. ORF X differed considerably in size and sequence between strains. A PCR-based assay was developed for early detection of CSSV in cacao plants [66]. In addition to the episomal virus, Geering *et al.* [7] reported the occurrence of an endogenous virus belonging to the newly proposed genus *Florendovirus* in the cacao plant. Jacquot *et al.* [117] reported agro-inoculation of CSSV using 1.2 unit length of CSSV genome in cocoa plants and observed that CSSV virions were seen only in the cytoplasm of phloem companion cells and a few xylem parenchyma cells. Light microscopy reported that the stem swelling resulted from a proliferation of xylem, phloem, and cortex cells. Quainoo *et al.* [118] reported a somatic embryogenesis-based method for elimination of CSSV from infected trees. CSSV was found in all the callus tissues induced by infected plants. The virus was transmitted to primary somatic embryos at the rate of 14%–19%. The virus-free primary somatic embryos from infected callus tissue converted into plantlets tested negative for CSSV.

### 7.4. Canna yellow mottle virus (CaYMV)

*Canna yellow mottle virus* (CaYMV) causes yellow mottle disease of canna (*Canna indica*), characterized by vein necrosis and mottling combined with streaking of stem and flowers. The disease was originally reported in Minnesota, Florida, and Washington, USA [119,120] and later in China, Japan, Italy, and the Netherlands [121,122]. A majority of diseased plants remain symptomless, and association of *Cucumber mosaic virus* and an unidentified flexuous rod-shaped virus was reported in a few cases. The causal virus was identified based on electron microscopy, ISEM, PCR, and sequence analysis of a portion of the RT/RNaseH region [123]. Recently, association of CaYMV with yellow mosaic disease of the betelvine was reported from Uttar Pradesh, India [124].

### 7.5. Citrus yellow mosaic virus (CYMV)

Ahlawat *et al.* [125] first reported the association of a badnavirus with the disease, which was later named *Citrus yellow mosaic virus*, based on particle morphology, serology, and partial nucleotide sequence of the viral genome. Citrus yellow mosaic (or citrus mosaic) disease has been reported so far only from India. It is common and especially severe in sweet orange (*Citrus sinensis*), with an incidence rate ranging from 10% to 70%. The reduction in fruit yield was 77%, and fruits from affected trees had 10% less juice and ascorbic acid [125]. The most characteristic symptoms of the disease are yellow mosaics of the leaves and yellow flecking along the veins. Grafting and dodder transmitted the virus to 14 citrus species and cultivars, including sweet orange, pumelo, Rangpur lime, Volkamer lemon, and sour orange. The virus is mechanically transmissible to *Citrus decumana*, Satgudi sweet orange, and pumelo. The first complete genome sequence of the virus from sweet orange and construction of an infective clone of CYMV for agroinfection was reported [38]. The CYMV genome consisted of 7559 bp with six ORFs on the plus strand of the genome, each capable of encoding a protein of >10kD (Figure 5). Phylogenetic analysis revealed that CYMV is most closely related to CSSV. Successful detection of CYMV in different citrus species and cultivars using PCR and nucleic acid spot hybridization assays were reported [126,127]. The complete genome sequencing of the virus isolates from *C. sinensis* (sweet orange*)*, *C. jambhiri* (rough lemon), *C. aurantifolia* (acid lime), and *C. grandis* (pumelo) revealed high homology among all virus isolates [37,128]. Geering *et al.* [7] reported occurrence of endogenous *Florendovirus* in citrus plant.

### 7.6. Commelina yellow mottle virus (ComYMV)

*Commelina yellow mottle virus* (ComYMV) is the type member of the genus *Badnavirus*. It infects the monocot weed, *Commelina diffusa* (day flower). The complete genome of the virus consists of 7489 bp with the three typical badnavirus ORFs capable of encoding proteins of 23, 15, and 216 kD [11]. Antibodies raised against the C-terminus of ORF I protein detected a 20 kD virus-specific protein at the surface of virions. This association was confirmed by immunosorbent electron microscopy and immunogold labeling. An antiserum raised against the putative ORF II gene product detected a 15-kD virus-specific protein confirming that also this one is virion-associated [26]. Through mutation studies, Tzafrir *et al.* [129] demonstrated that the N-terminal region of ORF III is required for systemic movement but not for replication of ComYMV. A construct containing 1.3 copies of the ComYMV genome was infective when introduced into *C. diffusa* through *Agrobacterium*-mediated infection. Analysis of the viral transcript indicated that the virus encodes a single terminally redundant genome-length-plus-120-nucleotide transcript [11,129]. The ultrathin sections of infected plants showed the presence of tubular structures containing virions. The exterior of these tubules reacted with antibodies to ComYMV movement protein but not with antibodies to coat protein, indicating that they play a role in the cell-to-cell movement of virions [130], as reported for several other plant viruses [131].

Medberry *et al.* [132] reported that the ComYMV promoter drives expression of GUS in transgenic tobacco primarily in the phloem, the phloem-associated cells, and the axial parenchyma of roots, stems, leaves, and flowers including anthers. Compared with the duplicated CaMV 35S promoter, the ComYMV promoter is 30% as active in tobacco and up to 25% in maize suspension cells. A study involving the transgenic expression of GUS driven by the ComYMV promoter in oats revealed that GUS expression was primarily localized in the vascular tissues of shoots, leaves, floral bracts, and roots [133]. Deletion analysis and functional studies of the ComYMV promoter in transgenic tobacco revealed the region between −870 bp and −232 bp to be responsible for the main promoter activity and the region downstream of it for tissue- (phloem-) specific expression [134]. Matsuda *et al.* [135] reported that the ComYMV promoter drives companion cell-specific gene expression in the leaves, stems, and roots of transgenic tobacco, indicating its usefulness in studies on the functions of companion cells and also in engineering crops that produce certain gene products in those cells to block long-distance movement of pathogens.

### 7.7. Dioscorea bacilliform virus (DBV) (DBALV and DBSNV)

*Dioscorea bacilliform virus* (DBV) occurs commonly in several species of *Dioscorea* (Yam) in the southern hemisphere. The disease symptoms include chlorotic-mosaic on leaves, leading to reduced sugar formation and minimal starch storage, thus causing a significant reduction in tuber yield and quality. The virus is transmitted vegetatively and through mealybugs (*Planococcus citri)*. Badnaviruses were first reported in the yam in association with a flexuous virus, causing internal brown spot disease in *D. alata* and *D. cayenensis-rotundata* in the Caribbean [136]. The badnaviruses isolated from Nigerian *D. alata* were named “*Dioscorea alata bacilliform virus*” (DBALV). The complete genome sequencing of two isolates of DBALV from Nigeria comprised 7413 bp and 7415 bp each with three standard badnavirus ORFs potentially coding for proteins of 29.5, 25.2, and 228.3 kD [137]. Later, Seal and Muller [33] reported complete genome sequences of two isolates of badnavirus from *D. sansibarensis* from Benin with only 62% identity with DBALV, thus representing a new badnavirus species termed *Dioscorea sansibarensis bacilliform virus* (DBSNV). The two isolates of DBSNV are 7262 and 7276 bp long, with four ORFs each coding for proteins of 143, 126, 1887, and 95 amino acid [33]. In addition to the complete sequence of DBALV and DBSNV, many partial badnaviral sequences have been generated using badnavirus degenerate primers from yam germplasm. Studies on the genetic diversity of 19 yam badnavirus isolates by sequencing the RT/RNaseH coding region revealed a high variability among isolates, clustering with either DBALV or DBSNV. One of the isolates showed only 75%–77% identity with either DBALV or DBSNV, indicating occurrence of a third species [138]. Comparison of RT/RNaseH coding region led to Kenyon *et al.* [139] proposing that there are at least 11 yam badnavirus species in the Southern Pacific region alone. In later studies involving phylogeny and pair-wise sequence comparison of 121 yam partial reverse transcriptase sequences, 12 yam badnavirus species were distinguished, including DBALV and DBSNV, mentioned above [140]. Five of those, characterized by a wide host range, are of African origin, while the other ones are characterized by a limited host range and are of Asian-Pacific origin. ELISA and Southern hybridization studies involving large samples representing eight species of yam support the presence of endogenous DBV (eDBV) in *D. rotundata*, but not in *D. praehensilis*, *D. abyssinica*, *D. alata*, and *D. Trifida* [56]. Umber *et al.* [141] reported molecular characterization of eDBV sequences in the genome of the African yam of the *D. cayenesis–rotundata* complex that showed sequence originating from various parts of badnavirus genomes, resulting in a mosaic structure. They reported that eDBVs belong to at least four distinct badnaviruses indicating multiple, independent endogenization events. So far there is no report on whether eDBV can give rise to infective virus particles under abiotic stress conditions.

### 7.8. Fig badnavirus 1 (FBV-1)

*Fig badnavirus 1*(FBV-1), causing mosaic disease in figs (*Ficus carica*), has been detected by PCR and RCA in 19 countries in Europe, Australia, Africa, and South and North America [55,142]. The virus can be mechanically transmitted to several herbaceous hosts. Based on the complete genome sequence, FBV-1 was identified as a distinct species of badnavirus. The whole genome showed that it contains 7140 bp with four ORFs encoding proteins of 15.3, 16.5, 212.5, and 17.0 kD. The key badnavirus motifs, with the exception of the protease motif, were found in the 212.5kD long ORF-derived polyprotein. The virus showed a close relationship to CSSV and CYMV [55]. Based on Southern hybridization, RT-PCR, and RCA, the virus was shown to be present both as episomes and integrated into the fig genome (eFBV-1). However, no information is yet available on whether eFBV-1 can be activated to give rise to infective FBV-1 under stress conditions.

### 7.9. Gooseberry vein banding associated virus (GVBaV)

Gooseberry vein banding disease (GVBD) occurs in cultivars of *Ribes* in Europe and North America. The diseased leaves show chlorotic laminar tissue adjacent to the central veins [65]. The causal virus, *Gooseberry vein banding associated virus* (GVBaV), is transmitted in a semi-persistent manner by different aphid species (*Aphis grossulariae*, *Nasonovia ribisnigri*, and *Hyperomyzus* spp.). *Ribes* spp. is vegetatively propagated, and hence the primary spread of the disease is by vegetative means. GVBD could be transmitted mechanically from infected gooseberry to *Nicotiana occidentalis* [65]. Xu *et al.* [143] reported full-length (7649–7663 bp) genomic sequences of four GVBaV isolates from different hosts and geographic regions. These isolates shared high identities (96.4%–97.3%). Phylogenetic analysis using the amino acid sequence of the ORF III derived putative protein showed that GVBaV groups most closely together with DBV, PVBV, and TaBV [143].

### 7.10. Grapevine vein clearing virus (GVCV)

*Grapevine vein clearing virus* (GVCV) is associated with severe vein-clearing and vine decline syndrome on grapevines (*Vitis vinifera*) in the Midwest region of the United States. The typical symptoms are translucent vein clearing on young leaves, short internodes, and decline of vine vigor. The disease could be transmitted through grafting. Sequencing of the complete virus genome revealed a 7753 bp sequence, coding for the three standard badnavirus ORFs [144]. Studies based on RT-PCR from infected plants clearly showed the presence of GVCV-specific transcripts in virus-infected plants. In addition to the episomal viruses, Geering *et al.* [7] reported the occurrence of an endogenous virus belonging to the newly proposed genus *Florendovirus* in grape. Further, they reported that in grapes, 9% of the endogenous florendovirus loci are located within introns and therefore may influence host gene expression.

### 7.11. Kalanchoë top-spotting virus (KTSV)

The top-spotting disease of kalanchoë (*Kalanchoë blossfeldiana*) is characterized by the appearance of numerous yellow spots on the leaves of affected plants. The disease occurs in many commercially traded cultivars of *K. blossfeldiana* in Europe and North America and can be economically damaging. The disease was shown to be graft-, seed-, and pollen-transmitted. It can also be transmitted by mechanical inoculation and by the citrus mealybug, *Planococcus citri.* In commercially grown kalanchoë, virus spread occurs primarily by vegetative propagation of infected stock plants [145].

The KTSV genome is 7591 bp long and contains the three typical badnavirus ORFs. Several oligonucleotide primer pairs, based on the KTSV genomic sequence, were used to efficiently detect the virus by screening of kalanchoë propagating stock and breeding lines. Two KTSV sequences, one symptom-inducing and the other not, were identified and differentiated by PCR amplification and digestion of the resulting amplicons with restriction enzymes. Preliminary results from graft-transmission tests and PCR indexing suggest that the non-symptomatic form of KTSV may represent an integrated viral element [58].

### 7.12. Pagoda yellow mosaic associated virus (PYMAV)

*Pagoda yellow mosaic associated virus* (PYMAV) was reported for the first time in China. It caused yellow mosaic disease of pagoda trees (*Styphnolobium japonicum*) in 2013. The complete genome of the virus determined by high throughput sequencing of siRNAs comprised 7424 bp, with five ORFs sharing 40%–45% identity with other badnaviruses [36]. PYMAV together with GVBV and GVCV formed a separate group that is distinct from other badnavirus groups. The PYMAV-specific viral siRNAs were mainly 21 nucleotides in length and distributed along the full genome of the virus.

### 7.13. Pineapple bacilliform comosus virus (PBCOV) and Pineapple bacilliform erectifolius virus (PBERV)

By using ISEM, *Pineapple bacilliform virus* was first detected in pineapple hybrids in Australia during 1995. It is transmitted by the gray pineapple mealybug, *Dysmicoccus neobrevipes*, and serologically related to SCBV [146]. An unidentified pineapple badnavirus was also observed in Hawaii [147]. Because of the ubiquity of the virus in both healthy and diseased plants, it is unlikely that badnavirus is the primary cause of the pineapple mealybug wilt disease [148]. Two badnaviruses, namely *Pineapple bacilliform comosus virus* (PBCOV) and *Pineapple bacilliform erectifolius virus* (PBERV), were reported from Australia [148]. In addition to these, an endogenous badnavirus (Pineapple pararetrovirus 1) and a retrotransposon (*Anannus metavirus* (AMtV) were found to be associated with pineapple mealybug wilt disease. The complete genome sequences of isolates of the virus both from China and from Hawaii comprised 7451 bp with the three standard badnavirus ORFs [149]. Putative endogenous pararetrovirus sequences were also identified in the pineapple [150].

### 7.14. Piper yellow mottle virus (PYMoV)

*Piper yellow mottle virus* (PYMoV) was first reported in 1997 in black pepper and betel vine in Malaysia, the Philippines, Sri Lanka, and Thailand [151]. Symptoms of the disease vary widely and are influenced by the cultivars and environmental conditions. The typical symptoms include chlorotic mottling, chlorosis, vein clearing, leaf distortion, reduced plant vigor, and poor fruit set. Surprisingly and in contrast to other cases, enhanced symptom expression was seen on plants continuously exposed to high temperatures (about 35 °C) and later became indiscernible when plants were continuously grown at a lower temperature (22–28 °C). When asymptomatic plants grown at 22–28 °C were transferred to 35 °C, there was a significant increase in both symptom severity and concentration of virus within 15–30 days [152]. The occurrence of PYMoV was also reported from India in black pepper, betel vine, and Indian long pepper [153,154,155] and recently also in many other *Piper* species [156].

The primary spread of the virus occurs through vegetative means and seeds [20,21], while secondary spread in the field is through different species of mealybugs, such as *Planococcus citri* and *Ferrisia virgata* [151,153], and by the black pepper lacewing bug (*Diconocoris distanti*) [157]. The first complete genome sequence of an isolate of PYMoV from India revealed 7622 bp with four ORFs [30]. ORFs I, II, and IV of PYMoV are reported as hypothetical proteins of unknown function with a predicted molecular mass of 15.7 kD, 17.1 kD, and 17.9 kD, respectively, while ORF III encodes the badnavirus standard polyprotein of 218.6 kD. Recently, complete genome sequencing of three more isolates of PYMoV (genome size ranging from 7559 to 7584 bp), infecting black pepper, betel vine and Indian long pepper revealed high homology among isolates (89% to 99%) [158]. PYMoV-free cuttings are important for planting of black pepper, a perennial, vegetatively propagated crop. Due to the symptomless nature of certain infected plants, sensitive assays such as PCR, real-time PCR, and loop-mediated isothermal amplification (LAMP) were developed for identification of virus-free mother plants for propagation [69,70,157].

### 7.15. Rubus yellow net virus (RYNV)

*Rubus yellow net virus* (RYNV), infecting *Rubus* species and its cultivars mainly in North America and Europe, is an essential component of the raspberry vein banding mosaic disease complex, which causes a serious decline in plant vigor and productivity. The virus is transmitted in a semi-persistent manner by the large raspberry aphid, *Amphorophora idaei*, in Europe and *A. agathonica* in North America. The virus-infected plants are either symptomless or show a very faint vein netting in the leaves. Virus-specific primers have been designed to detect the presence of the virus in infected plants [159]. The complete genome sequencing of an isolate of RYNV from Alberta, Canada revealed it is 7932 bp and seven ORFs [41] and that it most closely resembles the sequence of GVBV. In addition to the standard badnavirus proteins encoded by ORFs I, II, and III, proteins of 15 and 17 kD can be predicted from ORFs IV and V on the sense strand and proteins of 16 kD from both ORFs VI and VII, on the antisense strand (see Figure 5). The raspberry and strawberry plants agro-inoculated with more than a full-length genomic clone of the virus could be detected 14 days post-inoculation through an immunocapture assay. Small RNA sequence profiling of RYNV-infected red raspberry leaf tissue yielded a highly uneven genome-wide distribution of virus-derived siRNAs (vsiRNAs), with strong clustering at small defined regions distributed over both strands of the RYNV genome indicating that the raspberry interfering RNA pathway targets specific areas of the virus sequence, possibly as an antiviral defense mechanism [41]. Alternatively, those RNAs might represent decoys, as reported for CaMV and RTBV [160,161].

### 7.16. Schefflera ringspot virus (SRV)

Schefflera ringspot virus (SRV) infects Brassaia actinophylla (umbrella tree), Schefflera arboricola (dwarf umbrella tree) and Aralias (Polyscias balfouriana, P. balfouriana ‘Marginata’, P. fruticosa and P. guilfoylei) in Australia, Barbados, Cuba, Mauritius, Honduras, Taiwan, Thailand, and the USA [162]. SRV causes leaf mottling and chlorotic and necrotic ring spots in Schefflera, and chlorotic spotting, vein-clearing, and reduction of leaf size in Aralia. The virus is transmitted by the mealybug Planococcus citri. In ISEM tests, bacilliform virions occurring in schefflera and aralia were trapped by antisera to BSV, CSSV, RTBV, and SCBV. Based on particle morphology, genome properties, serological relatedness, and mealybug transmissibility, the causal virus was concluded to be a member of the genus, Badnavirus.

### 7.17. Spiraea yellow leaf spot virus (SLSV)

*Spiraea yellow leaf spot virus* (SLSV) causes yellow leaf spot disease of spiraea in the mid-western USA. The virus has a 7.4 kb dsDNA genome and resembles badnaviruses in particle and genome properties. Exceptionally, this virus is transmitted by an aphid, *Aphis spiraecola* [163].

### 7.18. Sugarcane bacilliform virus (SCBV) (SCBIMV and SCBMOV)

*Sugarcane bacilliform virus* (SCBV) was first observed in Cuba in 1985, and then in sugarcane in many countries around the world [164]. It causes chlorosis, mottling, and leaf freckle symptoms, but many of the infected plants are symptomless. Decreased juice, sucrose content, gravity, purity, and stalk weight were observed in SCBV-infected sugarcane in China [165]. It is postulated that the virus originated at the center of sugarcane origin in Papua New Guinea and spread to other cultivating areas with the movement of germplasm, especially noble canes. Further dissemination has been made by its mealybug vector *Sacharicoccus sachhari*. SCBV can be transmitted by *S. sacchari* from infected sugarcane to bananas, with SCBV-infected bananas developing symptoms indistinguishable from those described for BSV under laboratory conditions; however, natural infection of SCBV in bananas in field conditions has not been reported. A Moroccan strain of BSV is serologically indistinguishable from some isolates of SCBV. In laboratory experiments, SCBV (and BSV) can also be transmitted mechanically to healthy sugarcane and bananas [166]. Based on the PCR assay, Braithwaite *et al.* [63] reported the occurrence of SCBV in noble canes (*Saccharum officinarum*), commercial hybrids, and clones within the ‘Saccharum complex’ including *S. robustum*, *S. spontaneum*, *S. barberi*, *Erianthus arundinaceus*, and *E. ravennae*. Singh *et al.* [167] found mixed infection of SCBV with other viruses of sugarcane in India through PCR.

The first complete genome sequencing of the virus (Morocco isolate; now known as SCBMOV) revealed a genome of 7568 bp with the three standard ORFs and intergenic region [168]. A construct containing 1.1 copies of the cloned SCBMOV was infective to both rice and bananas by agroinoculation [168]. Complete genome sequencing of an Australian isolate (Ireng Maleng isolate) of SCBV (now known as SCBIMV) revealed a genome of 7687 bp [169]. The genomes of SCBMOV and SCBIMV share only 72% nucleotide sequence identity and are therefore considered by ICTV as two distinct species.

Based on partial SCBV sequences representing RT/RNase H domain of 35 isolates from Guadeloupe, Muller *et al.* [170] reported high molecular variability in the SCBV genome, distinguishing seven phylogenic groups named A to G including SCBMOV (group E) and SCBIMV (group F). SCBV groups A, B, C, and D belonged to badnavirus group 1, while SCBV groups E, F, and G belonged to badnavirus group 3, referring to the groups of badnavirus analyzed [95]. Based on the 80% nucleotide identity threshold in the RT/RNaseH domain, SCBV isolates of group A–B, C, and D are members of three additional SCBV species. Of these, complete genome sequences of two isolates of group A (7444 bp and 7446 bp) and one isolate of group D (7317 bp) are tentatively named as Sugarcane bacilliform Guadeloupe A virus and Sugarcane bacilliform Guadeloupe D virus [170]. Based on the complete genome sequencing of five isolates of SCBV from India (genome size ranging from 7568 to 7687 bp), Karuppaiah *et al.* [171] reported 69%–85% identities among the five Indian isolates that shared 70%–82% identity with SCBIMV and SCBMOV, indicating that the Indian SCBV isolates are distinct from other SCBV isolates. Phylogenetic analysis based on the partial RT/RNase H sequence identified three new species from India, for which the names Sugarcane bacilliform black Reunion virus (SCBBRV), Sugarcane bacilliform BO virus (SCBBOV), and Sugarcane bacilliform BB virus (SCBBBV) were proposed [171].

Southern analysis of infected plants of *Saccharum officinarum* indicated that SCBV is not integrated into the sugarcane genome [172]. They reported, however, the occurrence of SCBV DNA molecules larger than the unit length of 7.6 kb, possibly concatemers formed during replication, in infected plants and virions. However, Cai *et al.* [173] reported, based on PCR amplification and Southern hybridization, integration of SCBV DNA fragments into the genomes of *Saccharum* inter-specific hybrids (var ROC 205) grown in China. The variable region from SCBV was shown to serve as a promoter for high-level expression of foreign genes in both monocot and dicot transgenic plants [174,175].

### 7.19. Sweet potato pakakuy virus (Sweet potato badnavirus a + Sweet potato badnavirus b)

Based on small RNA sequencing of a Peruvian sweet potato land race, Kreuze *et al.* [176] reported the occurrence of two distinct badnaviruses, namely *Sweet potato badnavirus a* (SPVa) and *Sweet potato badnavirus b* (SPVb), which are collectively called *Sweet potato pakakuy virus* (SPPV). The complete genome sequence of both viruses was reported after filling the gap left in the sequence (obtained by small RNA sequencing) through PCR amplification. The complete genome of SPVa is 8082 bp and SPVb is 7961 bp long, both coding for four ORFs. Deep sequencing of small RNAs of symptomatic sweet potato plants from Honduras, Guatemala [177], and Tanzania [178] revealed the presence of the two viruses. A PCR-based method was developed for the reliable detection of both. A third SPV is indicated by a partial sequence (3065 nucleotides) encoding hypothetical movement and coat proteins, showing 86.3% and 73.1% identity to SPVb and SPVa.

### 7.20. Taro bacilliform virus (TaBV)

*Taro bacilliform virus* (TaBV) infecting taro (*Colocasia esculenta*) is distributed throughout the Pacific Islands. The disease is characterized by vein clearing, stunting, and downward curling of leaf blades. TaBV is transmitted by seeds and mealybugs (*Pseudococcus solomonensis*) [22,35,57]. The complete nucleotide sequence of an isolate from Papua New Guinea comprised 7458 bp with four ORFs. The size and organization of TaBV ORFs I to III are similar to the main ones of standard badnaviruses, while the locations of ORFs IV and X are similar to those of ORF IV of CYMV and ORF X of CSSV. Phylogenetic analysis showed that TaBV is most closely related to DBV [35]. Analysis of RT/RNaseH-coding region sequences of 22 TaBV isolates from Fiji, French Polynesia, New Caledonia, Papua New Guinea (PNG), Samoa, the Solomon Islands, and Vanuatu revealed up to 23% and 14% variability in nucleotide and amino acid sequences, while a variability of >33% was observed for the CP region [57]. Phylogenetic analysis of TaBV isolates from the Solomon Islands revealed the greatest and those from New Caledonia and PNG had the lowest variability. Based on sequences of the RT/RNaseH-coding region, a PCR-based diagnostic test that detects all known TaBV isolates was developed [57]. A sequence showing 50% nucleotide sequence identity to TaBV in the RT/RNase H region was also detected in all taro samples tested, indicating that this may represent either endogenous TaBV or the genome of an additional badnavirus. Recently, based on small RNA sequencing of symptomatic taro plants from China, Kazmi *et al.* [39] reported complete genome sequence of two isolates that shared nucleotide similarities of 44.1% to 55.8% with other badnaviruses (45.5% with TaBV), suggesting the occurrence of a new badnavirus species in taro, tentatively named *Taro bacilliform CH virus* (TaBCHV). The complete genome of two isolates of TaBCHV was 7641 bp long with six ORFs encoding proteins of 16.8, 14.1, 206.4, 13.2, 11.9, and 12.4 kD. A region of TaBV genome was shown to have promoter activity in taro leaf, banana suspension cells, and tobacco callus. When these promoters were evaluated in stably transformed, *in vitro*-grown transgenic banana and tobacco plants, all were found to drive near-constitutive expression of a reporter gene in the stem (or pseudostem), leaves, and roots, with the strongest expression observed in the vascular tissue, indicating that TaBV-derived promoters will be useful for the high-level constitutive expression of transgenes in either dicotyledonous or monocotyledonous species [179].

## 8. Putative Additional Species

### 8.1. Ambrosia asymptomatic virus 2 and Ambrosia asymptomatic virus 4

The occurrence of two distinct badnaviruses, namely *Ambrosia asymptomatic virus 2* and *Ambrosia asymptomatic virus 4*, was reported from the asymptomatic plants of *Ambrosia psilostachya* (western ragweed) from the USA. Nucleic acid isolated from virus-like particles prepared from asymptomatic plants, when amplified and sequenced, showed the presence of four viruses, including two badnaviruses [180].

### 8.2. Aucuba bacilliform virus (AuBV)

*Aucuba bacilliform virus* (AuBV) causes yellow ringspot or yellow mosaic in *Aucuba japonica*. The virus is transmitted by seeds and mealybugs.

### 8.3. Cycad necrotic leafspot virus (CyLNV)

*Cycad necrotic leafspot virus* (CyLNV) causes leaf necrosis disease of several cycads (*Ceratozamia, Stangeria* and *Zamia* spp.), characterized by chlorotic and necrotic leaf lesions that expand and produce larger necrotic zones. Electron microscopy of partially purified extracts showed 30 × 150 nm bacilliform virions that contained a ds DNA genome of 9.5 kb [181]. The complete genome of the virus from USA contained 9205 nucleotides with three ORFs.

### 8.4. Dracaena mottle virus (DrMV)

The mottle disease of *Dracaena* is characterized by mottling and chlorotic patches on the leaves of *Dracaena sanderiana* (known as “Fortune Bamboo”). The viral genome consists of 7531 bp and possesses seven putative ORFs on the plus-strand that potentially encode proteins of 17.6, 14.9, 215.0, 11.9, 11.3, 16.1, and 11.0 kD. ORF III of DrMV corresponds to that of badnaviruses. However, the nucleotide sequence coding for the RT and RNase H domain of DrMV shares less than 68% homology with that of any other known badnavirus. Southern hybridization of DNA from the infected plant indicated that the DrMV sequence is also integrated into the *D. sanderiana* genome [40].

### 8.5. Grapevine Roditis leaf discoloration-associated virus (GRLDaV)

*Grapevine Roditis leaf discoloration-associated virus* (GRLDaV) caused “Roditis leaf discoloration” (RLD), of grape vine, *cv.* “Roditis” in central Greece in the early 1980s [182]. RLD symptoms included yellow and/or reddish discolorations, malformation of the young leaves, and reduced size and low sugar content of the berries. siRNA sequencing from an infected vine showed the occurrence of a badnavirus of 6988 bp, including four ORFs. ORFs I, II, and IV code for proteins with unknown functions, while ORF III encodes a polyprotein with motifs related to the replication, encapsidation, and movement motifs of badnaviruses. Phylogenetic analysis showed FBV-1 as the closest to GRLDaV [32].

### 8.6. Mulberry badnavirus 1 (MBV-1)

*Mulberry badnavirus 1* (MBV-1) was reported to infect the mulberry (*Morus alba*) in Lebanon, showing symptoms of leaf mottling and vein yellowing. Typical badnavirus-like particles (150 × 30 nm) were seen in a partially purified preparation and tissue-thin sections of infected plants. The virus was also detected from mulberry samples collected from Turkey and Italy, though the majority of samples were free from symptoms. The complete genome of MBV-1, obtained through small RNA sequencing, contained all sequence features and the characteristic functional domains of the genus *Badnavirus*. The complete genome is 6945 bp long with two ORFs and shared high similarity (54%) with FBV-1. In contrast to badnaviruses, MBV-1 resembled the genome organization of PVCV (genus: *Petuvirus*), which has a single ORF [183,184].

### 8.7. Red clover bacilliform virus (RCBV)

*Red clover bacilliform virus* (RCBV) reported from *Trifolium pratense* plants showing dwarfing and mosaic symptoms in the Czech Republic showed bacilliform virions in phloem tissues that measured 220–500 nm × 30–31.5 nm. The virus was mechanically transmitted to *Nicotiana occidentalis* Wheeler, accession 37 B. The partial nucleotide sequence with similarity to open reading frame III of the badnavirus genome amplified had 74.4% nucleotide identity to that *of Ananas comosus* endogenous virus in the polyprotein gene covering reverse transcriptase [185].

### 8.8. Stilbocarpa mosaic bacilliform virus (SMBV)

The occurrence of *Stilbocarpa mosaic bacilliform virus* (SMBV) in *Stilbocarpa polaris* plants showing mild to severe yellow mosaic symptoms on subantarctic Macquarie island between Australia and Antarctica was observed in 1993 [186]. The cytoplasm of diseased samples contained bacilliform particles of varying sizes up to 100 nm in length and about 20 nm in diameter. DNA sequencing of the conserved reverse transcriptase region of the virus using degenerated badnavirus specific primers showed similarities with other badnaviruses, especially to badnaviruses from pineapple and sugarcane [186].

### 8.9. Yacon necrotic mottle virus (YNMoV)

*Yacon necrotic mottle virus* (YNMoV) infecting yacon (*Smallanthus sonchifolius*) in Korea was reported in 2013. The infected plants show necrosis, chlorosis, stunting, and malformation of leaves. Transcriptome sequencing of infected plants revealed the complete genome of the virus as 7661 bp in length with four ORFs, wherein ORF III showed 45% amino acid identity with that of FBV-1 [31].

### 8.10. Yucca bacilliform virus (YBV)

*Yucca bacilliform virus* (YBV) was first reported in 2001 in plants of *Yucca elephantipes* imported into New Zealand from Costa Rica and Guatemala [187]. The virus causes chlorotic lesions on the leaves along their length with increasing intensity towards the tips, which gradually turn necrotic with time. The causal virus was identified as a member of the badnavirus based on electron microscopy, PCR, and sequence analysis of the RT/RNase H region of the virus. Badnavirus DNA was also amplified from *Y. elephantipes* in New Zealand that were free of symptoms and virus particles, suggesting that YBV also occurs as an endogenous form in its host.

## 9. Conclusions and Future Research Needs

Badnaviruses are non-enveloped bacilliform DNA viruses with a monopartite genome. Currently, 32 species are confirmed, some of which are economically important in that they cause serious diseases in the tropics in crops such as banana, black pepper, cacao, citrus, sugarcane, taro, and yam. The majority of badnaviruses have a restricted host range. Many of the badnavirus-infected plants remain symptomless or show only mild symptoms. Masking of typical symptoms and their reappearance under abiotic stress are not uncommon. Hence the role of abiotic factors such as temperature, drought (soil moisture deficit stress), and nutrient stress in symptom development and the severity of the disease need to be studied in such badnaviruses. The possible role of biotic stress in badnavirus disease development and severity, as caused by other viral, fungal, bacterial, nematode, and insect pathogens, still needs to be studied. The genome length of badnaviruses varies between 7200 and 9200 bp with three to seven ORFs. Except for ORF III, which is a polyprotein, the functions of the other ORFs are still not known. Intergenic regions of the badnaviruses have the potential to be used as promoters and could be exploited in the expression of any foreign genes in plants.

Several of the pararetroviruses including badnaviruses occur also as endogenous viruses. The genus *Florendovirus* under the family Caulimoviridae has 34 distinct species known to exist only as endogenous viruses in a large number of plants. Of these, endogenous BSOLV, BSGFV, and BSIMV can give rise to episomal viruses in certain hybrids when subjected to one or the other abiotic stress, while endogenous viruses of other badnavirus reported so far are not known to give rise to infective episomal viruses under stress conditions.

PCR, IC-PCR, real-time PCR, LAMP, and RCA-based assays have been developed for the sensitive detection of a few badnaviruses. As PCR-based methods can give false positive results due to the presence of endogenous badnaviruses, there is an urgent need to develop other fool-proof methods to detect and distinguish episomal and endogenous badnaviruses in plants. Methods such as RCA and PCR are needed to detect plants that harbor BSV and eBSVs that can give rise to infective viruses, while RCA alone can identify virus-free plants that harbor non-infective endogenous viruses. This is important for the identification of virus-free plants to be used for propagation and also for international germplasm exchange. Though badnaviruses could be cured from the plant germplasm using cryopreservation, chemo-, and thermo-therapies combined with meristem tip culture, the success percentage is negligible. In the case of CSSV, somatic embryogenesis was used to obtain virus-free plants of cacao. These efforts must be pursued further for all badnaviruses for the production of certified virus-free plantlet production. Use of badnavirus-free planting material, providing required nutrients, cultural practices, and chemical measures for control of vector would help to manage the disease to a certain extent, but development of resistant cultivars would be the best option in developing IPM programs. So far those have not been reported. With the advent of genome editing tools like TALENS, Zinc finger nuclease, and the recent CRISPR-Cas9 with short guide RNA-based methods, it becomes possible to delete the unwanted integrated viral sequences, which can spontaneously give rise to episomal forms. Efforts to unravel the significance of integrated badnaviral sequences would lead to a better understanding of acquired immunity and natural RNAi in plant crops.

## Supplementary Materials

The following are available online at http://www.mdpi.com/1999-4915/8/6/177/s1, Table S1: Virus approved by ICTV (apr) (+) or tentative (−); endogenous sequences reported (End) (+); only partial sequence reported (#); Includes full genome sequence of two isolates (*).
