# Supplementary material for: Badnaviruses: The Current Global Scenario
Source: Viruses. 2016 Jun 22;8(6):177. doi: 10.3390/v8060177 (PMC4926197; doi:10.3390/v8060177)
Supplement: Supplementary file 1 [file viruses-08-00177-s001.docx]

Supplementary Material: Badnaviruses: Current Global Scenario

A.I. Bhat, T. Hohn, and R. Selvarajan

**Table S1.** Virus approved by ICTV (apr) (+) or tentative (−); endogenous sequences reported (End) (+); only partial sequence reported (^#^); Includes full genome sequence of two isolates (*).

| **Species** | **apr** | **Acronym** | **Host** | **Free** | **End.** | **Vector** | **Symptoms** | **Distribution** | **Collection** | **ORFs** | **Length (bp)** |
| --- | --- | --- | --- | --- | --- | --- | --- | --- | --- | --- | --- |
| *Aglaonema bacilliform virus* | + | ABV | *Aglaonema* spp. | + |  | unknown | Premature leaf senescence | North America |  |  |  |
| *Banana streak  GF virus* | + | BSGFV | *Musa* spp. cv.GoldFinger | + | + | mealybug | Chlorotic/necrotic leaf streaks | Africa, Asia, Pacific region | AY493509 KJ013507 | 3 | 7263 |
| *Banana streak  IM virus* | + | BSIMV | *Musa* spp. cv. Imove | + | + | mealybug | Chlorotic /necrotic streaks | Australia, Africa | HQ659760 HQ593112 KJ013508 | 3 | 7768-7769 |
| *Banana streak  MY virus* | + | BSMYV | *Musa* spp. cv. Mysore | + | + | mealybug | Chlorotic /necrotic leaf streaks | Africa, Asia and Pacific region | AY805074 EU140339 KJ013509 KF724854 KF724855 KF724856 KR014107 | 3 | 7641-7652 |
| *Banana streak  OL virus* | + | BSOLV | *Musa* spp. cv. Obino llwai | + | + | mealybug | Chlorotic /necrotic leaf streaks | Africa, Asia and Pacific region | AJ002234 JQ409539 JQ409540 KJ013506 | 3 | 7389 & 6950 |
| *Banana streak  UA virus* | + | BSUAV | *Musa* spp. | + |  | mealybug | Chlorotic /necrotic leaf streaks | Uganda | HQ593107 | 3 | 7519 |
| *Banana streak  UI virus* | + | BSUIV | *Musa* spp. | + |  | mealybug | Chlorotic /necrotic leaf streaks | Uganda | HQ593108 | 3 | 7458 |
| *Banana streak  UL virus* | + | BSULV | *Musa* spp. | + |  | mealybug | Chlorotic /necrotic leaf streaks | Uganda | HQ593109 | 3 | 7401 |
| *Banana streak  UM virus* | + | BSUMV | *Musa* spp. | + |  | mealybug | Chlorotic /necrotic leaf streaks | Uganda | HQ593110 | 3 | 7532 |
| *Banana streak  VN virus* | + | BSVNV | *Musa* spp. | + |  | mealybug | Chlorotic /necrotic leaf streaks | Vietnam | AY750155 KJ013510 | 3 | 7801 & 7797 |
| *Bougainvillea  spectabilis  chlorotic vein  banding virus* | + | BsCVBV | *Bougainvillea spectabilis* | + |  | unknown | Mottling, chlorosis, vein-banding, leaf distortion | Brazil, India, Taiwan | EU034539 | 4 | 8759 |
| *Cacao swollen  shoot virus* | + | CSSV | *Theobroma cacao* | + | + | mealybug, seed | Stem/root swelling, leaf mosaic and stunting | West Africa | L14546 AJ534983 AJ781003 AJ609020 AJ609019 JN606110 | 5 | 7006-7297 |

**Table S1.** *Cont.*

| **Species** | **apr** | **Acronym** | **Host** | **Free** | **End.** | **Vector** | **Symptoms** | **Distribution** | **Collection** | **ORFs** | **Length (bp)** |
| --- | --- | --- | --- | --- | --- | --- | --- | --- | --- | --- | --- |
| *Canna yellow  mottle virus* | + | CaYMV | *Canna* spp., *Piper betle* | + |  | unknown | Leaf and pseudo stem mottling | North America Japan, Netherlands, China, India | KT447043^#^ |  |  |
| *Citrus yellow  mosaic virus* | + | CYMV | *Citrus* spp. | + | + | mealybug | Yellow mosaic on leaves | India | AF347695 DQ875213 EU489745 EU489744 EU708317 EU708316 EU884191 EJ617224 IN006806 IN006805 | 6 | 7462-7559 |
| *Commelina yellow  mottle virus* | + | ComYMV | *Commelina diffusa* | + |  | mealybug, seed | Yellow leaf mottle | Caribbean Islands | X52938 | 3 | 7489 |
| *Dioscorea  bacilliform  AL virus* | + | DBALV | *Dioscorea alata* | + | + | mealybug | Chlorotic-mosaic on leaves | Africa, Asia, South and Central America and Oceania | X94575 to X94582* | 3 | 7413 & 7415 |
| *Dioscorea  bacilliform  SN virus* | + | DBSNV | *Dioscorea sansibarensis* | + | + | mealybug | Chlorotic-mosaic  on leaves | Africa, Asia, South and Central America and Oceania | DQ822073 DQ822074 | 4 | 7262 & 7276 |
| *Fig badnavirus 1* | + | FBV-1 | *Ficus carica* | + | + | unknown | Mosaic | Africa, Australia, Europe, South and North America | JF411989 | 3 | 7140 |
| *Gooseberry  vein banding  associated virus* | + | GVBaV | *Ribes* spp. | + |  | aphid | Chlorosis of leaves | Europe , North America | HQ852248 HQ852249 HQ852250 HQ852251 JQ316114 | 3 | 7649-7663 |
| *Grapevine vein  clearing virus* | + | GVCV | *Vitis vinifera* | + | + | unknown | Severe vein-clearing, vine decline | USA | JF301669 KJ725346 | 3 | 7753 & 7755 |
| *Kalanchoe  top-spotting virus* | + | KTSV | *Kalanchoe blossfeldiana* | + | + | seed, pollen, mealybug | yellow spots  on leaves | Europe and North America | AY180137 | 3 | 7591 |
| *Pagoda  yellow mosaic  associated virus* | + | PYMAV | *Styphnolobium japonicum* | + |  | unknown | Yellow mosaic | China | KJ013302 | 5 | 7424 |
| *Pineapple  bacilliform  CO virus* | + | PBCOV | *Ananas comosus* | + | + | mealybug | Mild mosaic or  no symptoms | Hawaii, China, Australia | GU121676 CQ398110 | 3 | 7543 & 7451 |
| *Pineapple  bacilliform  ER virus* | + | PBERV | *Ananas comosus* | + | + | mealybug | Mild mosaic or  no symptoms | Hawaii, China, Australia |  |  |  |

**Table S1.** *Cont.*

| **Species** | **apr** | **Acronym** | **Host** | **Free** | **End.** | **Vector** | **Symptoms** | **Distribution** | **Collection** | **ORFs** | **Length (bp)** |
| --- | --- | --- | --- | --- | --- | --- | --- | --- | --- | --- | --- |
| *Piper yellow  mottle virus* | + | PYMoV | *Piper* spp | + |  | mealybug black pepper lace bug, seed | Yellow mottle on leaves and stunting of plants | South Asia, Brazil | KC808712 KJ873041 KJ873042 KJ873043 | 4 | 7559-7584 |
| *Rubus yellow  net virus* | + | RYNV | *Rubus* spp. | + |  | aphid | Vein banding, mosaic | North America and Europe | KF241951 | 7 | 7932 |
| *Schefflera  ringspot virus* | + | SRV | *Shefflera* spp | + |  | mealybug | Ringspots, mosaic vein clearing | Worldwide |  |  |  |
| *Spiraea yellow  leaf spot virus* | + | SYLSV | *Spiraea* spp. | + |  | aphid | Yellow leaf spot  on leaves | North America | AF299074^#^ |  |  |
| *Sugarcane  bacilliform  IM virus* | + | SCBIMV | *Saccharum* spp., *Erianthus s*pp. | + | + | mealybug | Yellow leaf streaks | Australia, Guadeloupe, India | AJ277091 JN377536 | 3 | 7687 & 7673 |
| *Sugarcane  bacilliform  MO virus* | + | SCBMOV | *Saccharum* spp. *, Erianthus* spp. | + | + | mealybug | Yellow leaf streaks | Morocco, India, Cuba, Guadeloupe, China | M89923 JN377534 | 3 | 7568 & 7553 |
| *Sweet potato  badnavirus a and b* | + | SPV A  & B | *Ipomoea batatas* | + |  | unknown | Asymptomatic or mild symptoms | Honduras and Guatemala | FJ560943 FJ560944 | 4 | 8082 & 7961 |
| *Taro bacilliform  virus* | + | TaBV | *Colocasia esculenta* | + | + | mealybug, seed | Vein clearing, stunting, down curling of leaf blades | Pacific Island countries | AF357836 | 4 | 7458 |
| *Ambrosia  asymptomatic  virus 2* | − | AmbV-2 | *Ambrosia psilostachya* | + |  | unknown | Asymptomatic | USA | EU362853^#^ |  |  |
| *Ambrosia  asymptomatic  virus 4* | − | AmbV-4 | *Ambrosia psilostachya* | + |  | unknown | Asymptomatic | USA | EU362854^#^ |  |  |
| *Aucuba  bacilliform virus* | − | AuBV | *Aucuba japonica* | + |  | mealybug, seeds | Yellow ringpsot/  yellow mosaic | New Zealand |  |  |  |
| *Banana streak  Acuminata  Yunnan virus* | − | BSYUV | *Musa* sp. | + |  | unknown | Chlorotic /necrotic streaks | China | DQ092436 | 3 | 7722 |
| *Banana streak  CA virus* | − | BSCAV | *Musa* sp. | + |  | unknown | Chlorotic /necrotic streaks | Kenya, France | HQ593111 KJ013511 | 3 | 7408 & 7421 |
| *Banana streak  GD virus* | − | BSGDV | *Musa* sp. | + |  | unknown | Chlorotic /necrotic streaks | China | DQ451009 | 3 | 6950 |

**Table S1.** *Cont.*

| **Species** | **apr** | **Acronym** | **Host** | **Free** | **End.** | **Vector** | **Symptoms** | **Distribution** | **Collection** | **ORFs** | **Length (bp)** |
| --- | --- | --- | --- | --- | --- | --- | --- | --- | --- | --- | --- |
| *Banana streak  TRY virus* | − | BSTRYV | *Musa* sp. | + |  | unknown | Chlorotic /necrotic streaks | India | DQ859899 | 3 | 6950 |
| *Canna streak virus* | − | CaSV | *Canna indica* | + |  | unknown | Chlorotic/necrotic leaf streaks | North America | AJ810080^#^ |  |  |
| *Cycad necrotic  leafspot virus* | − | CyNLV | *Zamia* and *Ceratpzamia* spp | + |  | unknown | Chlorotic and necrotic leaf spots | North America | [EU853709](http://www.ncbi.nlm.nih.gov/nuccore/EU853709) | 3 | 9205 |
| *Dracaena  mottle virus* | − | DrMV | *Dracaena sanderiana* | + | + | unknown | Mottle and  chlorotic patches | China | DQ473478 EF494181 | 7 | 7531 & 7522 |
| *Grapevine Roditis  leaf discoloration- associated virus* | − | GRLDaV | *Vitis vinifera* | + | + | unknown | Yellow/reddish leaf discoloration | Greece | HG940503 | 4 | 6988 |
| *Mimosa  bacilliform virus* | − | MBV | *Albizzia julbrissin* | + |  | seed |  |  |  |  |  |
| *Mulberry  badnavirus 1* | − | MBV-1 | *Morus alba* | + |  | unknown | Leaf mottling and vein yellowing | Lebanon, Turkey, Italy | LN651258 | 2 | 6945 |
| *Pelargonium vein banding virus* | − | PVBV | *Pelargonium x hortorum* (Florist’s geranium) | + |  | seed | Chlorotic vein banding | USA | [GQ428155](http://www.ncbi.nlm.nih.gov/nuccore/GQ428155) | 3 | 7586 |
| *Red clover  bacilliform virus* | − | RCBV | *Trifolium pratense* | + |  | mechanical | Dwarfing  and mosaic | Czech Republic | JX069965^#^ |  |  |
| *Stilbocarpa mosaic  bacilliform virus* | − | SMBV | *Stilbocarpa polaris* | + |  | unknown | Mosaic | Subantarctic (Macquarie Island) | AF478691^#^ |  |  |
| *Sugarcane bacilliform GA virus* | − | SCBGAV | *Saccharum* hybrid | + |  | mealybug | Yellow leaf streaks | Guadeloupe | FJ824813 FJ824814 | 3 | 7444 & 7446 |
| *Sugarcane bacilliform GD virus* | − | SCBGDV | *Saccharum officinarum* | + |  | mealybug | Yellow leaf streaks | Guadeloupe | NC013455 | 3 | 7317 |
| *Sugarcane bacilliform BO virus* | − | SCBBOV | *Saccharum* hybrid | + |  | mealybug | Mild mosaic, freckles | India | JN377533 | 3 | 7826 |
| *Sugarcane bacilliform BB virus* | − | SCBBBV | *Saccharum officinarum* | + |  | mealybug | Mild mosaic, freckles | India | JN377535 | 3 | 7613 |
| *Sugarcane bacilliform BR virus* | − | SCBBRV | *Saccharum officinarum* | + |  | mealybug | Mild mosaic, freckles | India | JN377537 | 3 | 7884 |
| *Taro bacilliform  CH virus* | − | TaBCHV | *Colocasia esculenta* | + |  | unknown | Mild feathery mosaic and brown spots | China | KP710177 KP710178 | 6 | 7641 |
| *Turmeric mild  chlorosis virus* | − | TuMCV | *Curcuma longa* | + |  | unknown | Mild chlorosis/  mottling on leaves |  | AJ810082^#^ |  |  |
| *Yacon necrotic  mottle virus* | − | YNMoV | *Smallanthus sonchifolius* | + |  | unknown | Necrotic mottle,  chlorois, stunting,  leaf malformation | Korea | KM229703 | 4 | 7661 |
| *Yucca  bacilliform virus* | − | YBV | *Yucca elephantipes* | + | + | unknown | Mild chlorosis/  mottling on leaves | Central America and New Zealand | AF468688^#^ |  |  |
